# Post-exertional malaise in daily life and experimental exercise models in patients with myalgic encephalomyelitis/chronic fatigue syndrome

**DOI:** 10.3389/fphys.2023.1257557

**Published:** 2023-12-04

**Authors:** Nina K. Vøllestad, Anne Marit Mengshoel

**Affiliations:** Department of Interdisciplinary Health Science, Institute of Health and Society, University of Oslo, Oslo, Norway

**Keywords:** post-exertional malaise (PEM), myalgic encephalomyelitis/chronic fatigue syndrome (ME/CFS), exercise, cardiopulmonary exercise test (CPET), neuroendocrinological system, muscle weakness, adrenaline, noradrenaline

## Abstract

Post-exertional malaise (PEM) is commonly recognized as a hallmark of myalgic encephalomyelitis/chronic fatigue syndrome (ME/CFS) and is often used as one of several criteria for diagnosing ME/CFS. In this perspective paper we want to reflect on how PEM is understood, assessed, and evaluated in scientific literature, and to identify topics to be addressed in future research. Studies show that patients use a wide variety of words and concepts to label their experience of PEM in everyday life, and they report physical or mental exertions as triggers of PEM. They also report that PEM may have an immediate or delayed onset and may last from a few days to several months. When standardized exercise tests are used to trigger PEM experimentally, the exacerbation of symptoms has a more immediate onset but still shows a wide variability in duration. There are indications of altered muscular metabolism and autonomic nervous responses if exercise is repeated on successive days in patients with ME/CFS. The decreased muscular capacity appears to be maintained over several days following such controlled exercise bouts. These responses may correspond to patients’ experiences of increased exertion. Based on this background we argue that there is a need to look more closely into the processes occurring in the restitution period following exercise, as PEM reaches the peak in this phase.

## Introduction

Myalgic encephalomyelitis/chronic fatigue syndrome (ME/CFS) is a symptom-based diagnosis characterized by patient-reported inexplicable, incapacitating, persistent or relapsing fatigue, unrefreshing sleep, cognitive dysfunctions, and musculoskeletal pain that heavily impact patients’ lives (Nacul et al., 2021). Over the years several sets of diagnostic criteria have been developed to classify ME/CFS. Examples are the Oxford Criteria (Sharpe et al., 1991), the Fukuda Criteria (Fuku et al., 1994), and the International Consensus Criteria, ICC (Carruthers et al., 2011). Post-exertional malaise (PEM) was included in the CFS case definition by Fukuda and co-workers (Fuku et al., 1994) in 1994, although PEM was not explained nor further defined by these authors. In their criteria, PEM was one of nine symptoms to be considered for the diagnosis although PEM did not need to be present. In the International Consensus Criteria, however, PEM is considered a hallmark used to differentiate ME/CFS patients from patients with other chronic illnesses and persistent fatigue (Carruthers et al., 2011). These authors describe PEM as a perceived exacerbation of some, or all symptoms of ME/CFS after physical or cognitive exertion. Further, PEM is described as a disproportional worsening of symptoms due to physical or cognitive efforts that previously was well tolerated, typically with a delayed onset and lasting for variable and often extended periods of time (Nacul et al., 2021). In the later years, PEM has been investigated by qualitative interviews of patients, assessed by standardized self-reports alone or in combination with experimental triggering actions such as exercise tests or mental challenges (Nacul et al., 2021). However, to our knowledge the relationship between PEM as experienced in every-day life and experimentally induced PEM is scarcely examined.

On this background, the purpose of this perspective paper is to examine if there is coherence between PEM as described by patients with their own words, PEM assessed by standardized questionnaires, as well as exercise-induced PEM by experimental models. In addition, we will discuss how the exercise tests can be further developed to capture relevant aspects of PEM and the possible underlying biological mechanisms.

## PEM as experienced in everyday life

PEM is narrated by patients as well as assessed by structured self-reports. Moreover, PEM has been examined in terms of exacerbation of ME/CFS symptoms, what triggers the exacerbations in daily life, as well as the time of onset and duration of symptoms before recovery. The literature used is summarized in Table 1. PEM is found to relate to exacerbations of fatigue, cognitive difficulties (cannot think clearly, memory problems), sleep problems, neuromuscular complaints (pain/aches and weakness), bodily heaviness, flulike symptoms and impacts on daily functioning (Baraniuk et al., 2013; Chu et al., 2018; Jason et al., 2018; Davenport et al., 2023a). These findings were also supported by a focus group interview with patients (Stussman et al., 2020). It is noteworthy that high PEM also seems to associate with more symptoms, psychological distress, and social burden (May et al., 2020).

**TABLE 1:** Self-reported post-exertional (PEM) symptom in patients with myalgic encephalomyelitis/chronic fatigue syndrome (ME/CFS).

Authors	Purpose of the study	Case definition	Study design	Patients	PEM descriptions	PEM triggers	PEM onset and duration
Country							
Qualitative interviews							
Krabbe et al. (2022)	What do young women who are in recovery from ME/CFS tell about their experience of falling severely ill during childhood and adolescence?	Self-reported diagnosis set by a physician	Narrative qualitative interviews with overarching open-ended questions	13 women with ME/CFS during childhood or adolescence now in recovery or recovered	Gradually developing unhomeliness and feeling pushed toward the edge; and left abandoned on the sidelines		
Norway				Age range at time of interviews 16–30 years			
Parslow et al. (2018)	Describe adolescents with ME/CFS’ experiences of fatigue, symptom fluctuation	Diagnosis set in clinics by an experienced pediatrist	Qualitative semi-structured interviews	21 adolescents with ME/CFS and their parents	Fluctuations in fatigue and other symptoms create good and bad days. Worsening could appear for no reason and as a payback after activity; worded as “wiped out,” “absolute crash,” ‘knocked out,”, “zonked out,” and “out cold”		
UK				Age range 12–17 years			
Olson et al. (2015)	Explore the meaning of fatigue	Fukuda criteria	Ethnoscience interviews with overarching open-ended questions	13 women and 1 man	Tiredness expresses prior to illness, in remission, or “good days”. Fatigue describes ME/CFS on daily basis. Exhaustion expresses overexertion		
Canada				Age range 37–68			
Stussman et al. (2020)	Expand the understanding of PEM, its manner of onset, timeframes for onset, peak, duration	Diagnosed by physicians and referred to a cardio-pulmonary exercise test	Focus group interviews	43 subjects	PEM was described as “Flu-like” exhaustion, Cognitive problems of thinking clearly, finding words, and complete fog	Three broad categories of activity: physical activity, cognitive effort, and emotional moments. The threshold for activity depends on their baseline	After daily activities: onset after 12–48 h, peak 48 h, duration 2–7 days
USA			Had undergone cardio-pulmonary exercise test	21% men	Neuromuscular complaints as pain/aches and weakness		After exercise test: immediate onset, peak 24 h, duration 72 h
				Age range 30–69 years			
Structured questions with open ended responses							
Hartle et al. (2021)	Examine patients’ responses on PEM triggers, experiences, recovery, and prevention	Fulfilling at least one of diagnostic criteria: Fukuda, International Consensus Criteria, Canadian Consensus Clinical Criteria	Online survey of PEM questionnaire designed for the study with 4 items with open-ended responses and 2 with predefined alternatives	75 subjects sick ≥4 years and 76 sick ≥10 years	Fatigue 21% of the symptoms (e.g., weakness, heavy limbs, flu-like symptoms, feeling ill), pain/musculoskeletal symptoms 18%, orthostatic intolerance 14%	Most common exertion induced by medium levels of physical (71%) and cognitive activities, at any levels these two factors accounted for 89%	Onset: Within minutes 35%
US				Age range 18–65 years	Rest used as strategies for recovering, and pacing for prevention	Stress by those <4 years sick (19%)	Within hours 40%
				26% men			Within ≥ 1 day 25%
							Duration: At least 1 day 31%
							Several days 48%
							At least 1 week 13%

The US Committee on the Diagnostic Criteria for ME/CFS reports that patients typically call PEM a “crash,” “flare-up,” “collapse,” “debility” or “set-back” (Locher and IOM, 2015). In a similar vein, two qualitative studies showed that patients express their experience in terms of “wiped out”, “absolute crash,” “knocked out,” “zonked out” (Parslow et al., 2018), or a “bodily lock down” (Krabbe et al., 2022). A survey of international respondents showed that the most preferred wordings for PEM were “crash” and “exhaustion” (Jason et al., 2018). Interestingly, a Canadian study showed that patients themselves discriminate fluctuations in ME/CFS by using the words of tiredness, fatigue, and exhaustion (Olson et al., 2015). Tiredness was used to describe experiences prior to the diagnosis or on good days (i.e., what they find normal and healthy), whereas fatigue referred to how it was like on daily basis with ME/CFS. In contrast, exhaustion described the experiences after overexertion, i.e. related to PEM.

A frequently applied questionnaire for assessing symptoms among patients with ME/CFS is the DePaul Symptom Questionnaire (DSQ) (Jason and Sunnquist, 2018). Based on the five PEM items from DSQ and five additional questions, Cotler and coworkers developed a scale to identify the presence of PEM (Cotler et al., 2018). Respondents are asked to rate how bothersome and frequent the following symptoms are: “Dead, heavy feeling after starting to exercise,” “Next day soreness or fatigue after non-strenuous, everyday activities,” “Mentally tired after the slightest effort,” “Minimum exercise makes you physically tired,” “Physically drained or sick after mild activity” (Cotler et al., 2018). Thus, there is a discrepancy between wordings used by patients to describe PEM and those used in items of the standardized questionnaire. ”Dead, heavy feeling” may better be captured by “flu-like” or “crash” experience used by patients. Moreover, “tiredness” and “fatigue” in the questionnaire differ from the term “exhaustion” used by patients to describe PEM. Furthermore, non-strenuous everyday activities and minimum exercise are rather unspecific terms. Neither of the terms necessarily discriminate between daily activities and an extraordinary triggering event. Although, the DSQ is found to have a good internal consistency (Brown and Jason, 2014; Jason et al., 2015a), one may question the items’ face validity since there are deviations between the expressions used by patients in qualitative studies and in questionnaires.

Focus group interviews have outlined that a broad set of non-strenuous daily activities may trigger PEM, such as household chores, social activities, physical exercise, cognitive activities, and emotional moments (Stussman et al., 2020). Another qualitative study also underscores that all kinds of activities can lead to a “payback time” the following days, and these authors identified also a symptom fluctuation unrelated to triggers, portrayed as a state where ME/CFS operates “on its own” (Parslow et al., 2018). A quantitative study categorized the triggers as emotional stress, activities in daily life, positional changes, noise, and other sensory overloads (Holtzman et al., 2019). Engagement in high-strenuous activities can also be accompanied by discomfort in terms of fatigue and muscle soreness among healthy people. However, for patients with ME/CFS, this seems to occur even after low-strenuous physical and cognitive activities (Holtzman et al., 2019; Hartle et al., 2021; Davenport et al., 2023a). Moreover, patients also describe a symptom fluctuation over time displayed as “booms and busts” (Strassheim et al., 2021) or as a gradual down-spiraling deterioration process accompanied by an increasing intolerance of triggers (Krabbe et al., 2022). It thus seems to be good coherence between freely voiced triggering experiences and assessments of triggers.

A study of 704 respondents examined whether PEM was a unified entity or composed of different constructs (McManimen et al., 2019). A factor analysis of a large set of symptoms combined with triggers demonstrated that PEM is composed of two different constructs: generalized fatigue and muscle-specific fatigue. The first comprised symptoms of more generalized feeling of physical and mental fatigue, whereas the muscular construct included symptoms that referred to pain, weakness or fatigue in muscles following exertion. This finding may have important implications, particularly considering the various types of triggers reported. An interesting question is whether the two components of PEM are connected to specific and different triggers.

It is also a question whether there is a typical timeline for when PEM begins, reaches a peak and subsides. The US Committee on the Diagnostic Criteria for ME/CFS describes PEM as having an immediate onset or occurring within 30 h, but it is outlined that PEM may also develop hours or days after the trigger has ceased (Locher and IOM, 2015). Moreover, the committee describes an unpredictable duration of PEM as it may last for hours, days, weeks, and even months. In accord, a survey demonstrated a large variability in reported onset and duration of PEM, and most of the respondents reported that they sometimes experienced an immediate onset and sometimes a delayed onset (Holtzman et al., 2019). The delay ranged from 1 h to a week, and the duration could last from 1 day to several months. Similar results have been reported by others (Chu et al., 2018; Stussman et al., 2020). Thus, the onset and duration of PEM have no definite pattern and seem to vary both within and between patients.

## PEM in relation to experimental exercise tests

Due to the lack of objective signs or a clear and uniform picture of how the patients present PEM and in what triggers PEM, standardized tests have been employed and recommended for diagnostic purpose (Nacul et al., 2021). These tests may be important tools not only in the diagnostic process, but also in generating scientific knowledge that may lead to a shift from symptom-based to biology-based diagnosis. Applying exercise protocols for research purposes may help us to understand biological underlying mechanisms for PEM. This approach may be valuable for diagnostic and research purposes, although for instance the NICE guidelines recommend caution in applying exercise as part of management (NICE, 2021). The controlled conditions of the experimental tests with a follow-up over time, is in keeping with diagnostic recommendations (Nacul et al., 2021).

The exercise tests have several important qualities, including standardization of workload and the possibility of matching patients and healthy controls with regard to differences in for instance gender, age, and physical fitness. Furthermore, the exercise tests can require a workload beyond the physical demand of daily activities. The symptoms induced by exercise will thus reflect a response to an extraordinary and a well-defined physical exertion. We will below present and discuss exercise-induced symptoms when exercise is used as a trigger and compare the responses during and after exercise seen in persons with ME/CFS with healthy controls. Three relatively recent systematic reviews and meta-analyses will be used (summarized in Table 2) together with additional studies published more recently.

**TABLE 2:** Responses to exercise in persons with ME/CFS or long Covid compared with controls. Summary of systematic reviews and meta-analyses.

Authors	Main purpose and methodology	No included studies and no of participants included in meta-analysis	Types of exercise included	Main results
Country				
Barhorst et al. (2020)	Quantify the perceived exertion (RPE) response to acute aerobic exercise involving people with ME/CFS compared with healthy controls	37 studies	Cycle ergometer—continuous increasing (CPET)- from low to high intensity	Elevated RPE in people with ME/CFS during exercise
	Systematic review and meta-analysis	1,086 patients with ME/CFS	Treadmill—continuous increasing- from low to high intensity	
		686 healthy controls	Combined arm-leg ergometer—continuous steady—moderate intensity	
			Cycle ergometer—continuous steady—moderate intensity	
Loy et al. (2016)	Estimate the effect of completing a single bout of exercise on fatigue states in people with ME/CFS compared with health controls	7 studies	Cycle ergometer—continuous increasing (CPET)- from low to high intensity	During exercise fatigue increases more in people with ME/CFS than in healthy controls
	Systematic review and meta-analysis	159 patients with ME/CFS	Treadmill—continuous increasing- from low to high intensity	The effects were heterogeneous between studies
		Number of healthy controls not provided	Arm-leg ergometer—continuous steady—moderate intensity	The largest difference was seen 4 h or later after exercise
			Combined arm-leg ergometer—continuous steady—moderate intensity	
			Treadmill—continuous steady—moderate intensity	
			Handgrip—repeated—moderate	
Barhorst et al. (2022)	Quantify the effect of a single aerobic exercise bout on pain symptom severity in people with ME/CFS and FM	15 studies	Cycle ergometer—continuous increasing (CPET)- from low to high intensity	People with ME/CFS and FM experience small to moderate increases in pain symptom severity after exercise
	Systematic review and meta-analysis	306 patients with ME/CFS	Combined arm-leg ergometer—continuous steady—moderate intensity	
		292 healthy controls	Cycle ergometer—continuous steady—moderate intensity	
			Treadmill—continuous steady—moderate intensity	
			Arm ergometer—continuous—high intensity	
Nelson et al. (2019)	To determine whether there were differences in heart rate parameters between patients with ME/CFS and healthy controls	24 studies^a^	Cycle ergometer—continuous increasing (CPET)- from low to high intensity	Heart rate parameters during exercise differed between people with ME/CFS and healthy controls, indicating reduced vagal and increased sympathetic modulation of heart rate
	Systematic review and meta-analysis	1,129 patients with ME/CFS^a^	Treadmill—continuous increasing- from low to high intensity	
		626 healthy controls^a^	Cycle ergometer—continuous steady—moderate intensity	
			Treadmill—continuous increasing- from low to moderate intensity	
Durstenfeld et al. (2022)	To estimate the difference in exercise capacity between individuals with and without long COVID symptoms and to elucidate possible mechanisms	3 studies	Cycle ergometer (upright or supine)– continuous increasing (CPET)- from low to high intensity	Aerobic capacity was lower in individuals with long COVID symptoms
	Systematic review and meta-analysis	1,228 individuals with long COVID	Treadmill—continuous increasing- from low to high intensity	Several factors may contribute to decreased aerobic capacity, including deconditioning
		932 individuals without long COVID symptoms		

^a^ Only studies and participants studied with exercise models.

ME/CFS, Myalgic encephalomyelitis/chronic fatigue syndrome.

FM, fibromyalgia.

CPET, cardiopulmonary exercise test.

The most frequently self-reported responses to exercise in ME/CFS patients, are perceived exertion, fatigue and pain (Table 2). Ratings of perceived exertion (RPE) reflects the person’s evaluation of effort during exercise, whereas fatigue will here be understood as the subjective experience in the period following exercise. In a review and meta-analysis of 15 studies, Barhorst and co-workers showed that patients with ME/CFS perceived exercise as more effortful than healthy controls based on the ratings of perceived exertion (RPE) during exercise (Barhorst et al., 2020). This difference remained when controlling for confounding and moderating factors. It is important, however, that only one of the included studies had physical fitness as one of the matching criteria (Oosterwijck et al., 2017). More recently, a higher RPE reported by patients was also observed after matching patients and controls for fitness to achieve comparison at similar relative workload (Cook et al., 2022). Hence, the literature is quite consistent in reporting that patients with ME/CFS report higher exertion of effort during exercise than healthy controls, even when they do not differ in physical fitness.

Another systematic review and meta-analysis by Loy and co-workers examined the changes in fatigue after exercise (Loy et al., 2016). Even though the number of studies and participants was relatively low, they found that exercise triggered a larger increase in fatigue after exercise among patients with ME/CFS compared with controls. The group differences were most pronounced when fatigue was measured 4 h or later after end of exercise. Furthermore, enhanced fatigue was observed following different types of exercise (e.g., intermittent bouts or continuous) and workload (submaximal and maximal) (Sandler et al., 2016). A recent study examined the temporal pattern following two exercise bouts separated by 24 h (Moore et al., 2023). Patients with ME/CFS reported increased fatigue reaching a peak approximately 2 days after the last exercise bout. In contrast, fatigue remained low without any exacerbation among the healthy controls in the post-exercise period. In another recent study Davenport and co-workers found a prevalence of increased fatigue in approximately 60%–65% of the patients with ME/CFS 1 week after exercise (Davenport et al., 2023b). There appears to be a consistent finding that patients with ME/CFS differ from healthy controls in developing a long-lasting fatigue following exercise, and that patients need many days to recover to pre-exercise fatigue.

In addition to fatigue and perceived exertion, exercise may induce a substantial increase in pain intensity. A systematic review and meta-analysis from 2022 by Barhorst and co-workers reported that exercise triggered higher pain intensity in patients with either fibromyalgia or ME/CFS compared with healthy controls (Barhorst et al., 2022). Five of the studies only included patients with fibromyalgia. Both patient groups showed a significantly higher pain response compared with the control groups. The largest differences in pain intensity were seen 1–3 days after exercise. Together with the recent finding of increased prevalence of pain in ME/CFS patients in the restitution phase following exercise (Davenport et al., 2023b), the review clearly indicates that exercise triggers an aggravated pain response in ME/CFS patients compared with healthy controls, and similar to that seen in patients with fibromyalgia.

Exercise may also trigger other symptoms, for instance discomfort, sleep disturbances, headache, and neurological symptoms (Sandler et al., 2016; Stussman et al., 2020; Davenport et al., 2023b). Some studies have also used more composite scales for assessment of multiple symptoms, or simply asked for the number of days to recover (Baraniuk et al., 2013; Strand et al., 2019; Hodges et al., 2020; Moore et al., 2023). The results of these studies further support that ME/CFS patients report a higher level of various symptoms post-exercise compared with healthy controls. Two studies examined the patients’ own assessment of recovery after the second day with exercise and reported a time for recovery varying from 6 to 12 days (Hodges et al., 2020; Moore et al., 2023). Also, the duration of aggravated symptoms varied from a few days and up to weeks. The variability in symptom duration may partly be due to different exercise models employed, but there seems to be an additional heterogeneity that has other causes.

## Biological responses to experimental exercise tests

The experience of PEM reported by patients with ME/CFS after exertion has led to a series of studies to examine possible underlying biological mechanisms and to identify leads for diagnostic criteria. Factors related to responses in the neuroendocrine and cardiovascular systems, as well as metabolism and muscular weakness have been main targets of research. The exercise models are of particular interest to use as triggers of potential biological mediators of the aggravated fatigability and slow restitution. One line of research has been to investigate deviations in the autonomic function, for instance as reflected in neuroendocrine responses. Another has been to examine metabolism and energy consumption (Lim et al., 2020). During exercise the pulmonary and cardiovascular systems need to adjust to match the energy demand of the workload. Hence, the biological responses can be studied in a controlled way and thereby allow comparisons between patients and healthy controls.

A commonly used standardized exercise test is the cardiopulmonary exercise test (CPET) which provides a measure of functional capacity and indicators of how the body adjust and adapt to the increased metabolic demand. The test is typically performed on a cycle ergometer with stepwise increment in workload. Assessments include oxygen uptake, ventilation variables, heart rate, gas exchange, blood lactate and indicators autonomic responses (Lim et al., 2020). Oxygen uptake will increase as workload increases, until the cardiopulmonary system no longer can match the energy demand of the working muscles. With the use of cycle ergometers, the energy requirements will be determined by the chosen workload of the bike, and thus the same for all since the individual technique play a negligible role for this kind of exercise (Åstrand et al., 2003). Hence, at submaximal workload, when the cardiopulmonary system is able to provide sufficient amount of oxygen to the muscles, two persons exercising at the same absolute workload will have almost equal oxygen uptake. Other parameters may vary, such as frequency of ventilation and heart rate. At higher workloads, less fit individuals may approach or exceed their cardiopulmonary capacity to provide oxygen to the muscles and oxygen uptake will no longer be sufficient. Anaerobic energy sources will gradually be used, resulting in a steeper rise for instance in blood concentrations of lactate and CO_2_. These physiological responses form the basis for using CPETs to investigate deviations in the cardiopulmonary system or muscular metabolism (Adachi, 2017).

Although two persons have the same energy requirement and oxygen uptake at a given submaximal workload, a less fit individual with lower maximal aerobic capacity, will exercise at a higher relative intensity compared with his maximal capacity. Thus, exercise intensity is often expressed as the percentage of their maximal oxygen uptake. A higher relative workload is typically accompanied by for instance a higher ventilation rate, higher heart rate and higher lactate concentration in blood (Åstrand et al., 2003). Comparisons of various physiological responses to exercise must therefore be done at similar relative workload, or ensure a careful matching of cases and control, including physical fitness.

Unfortunately, differences in physical fitness often insufficiently taken into consideration in studies comparing responses to exercise in ME/CFS patients and healthy controls. Some studies include exercise frequency (e.g., less than twice a week (Lien et al., 2019) or less than 30 min per week (Davenport et al., 2023b) to reduce the potential effect of different fitness. However, as shown in the two reviews in 2020 and 2022 by Barhorst and coworkers, almost no studies matched the participants on physical fitness (Barhorst et al., 2020; Barhorst et al., 2022). Hence, the results are difficult to interpret, as inactivity can lead to considerable decline in physical fitness and altered responses during exercise (Saltin et al., 1968).

In 2022 Cook and co-workers published a paper which examined exercise responses in fitness-matched patients with ME/CFS and healthy controls (Cook et al., 2022). In this well-controlled study, they found that most differences between healthy and controls disappeared after matching for physical fitness. Of particular interest is the lack of differences in cardiometabolic responses. However, the patients reported a higher RPE and had a lower respiration frequency and higher tidal volume. These observations indicate that several of the differences previously reported during exercise, can be explained by lower aerobic fitness among patients with ME/CFS compared with controls.

Over the last decade, a particular interest has been given to an exercise model involving two CPETs repeated within 1–3 days. By introducing a second exercise bout in the recovery period from exercise at Day 1, the idea is that deviations in fatigability or underlying biological responses might be more prominent and thus detectable. One advantage is that in this model the responses to exercise on the second day are compared with each individual’s responses the first test day. To some extent, this reduces the problems with difficulty in matching on fitness level. Lim and co-workers reviewed the studies exploring CPET on repeated days in ME/CFS patients and controls (Lim et al., 2020). Although only five studies with 98 patients and 51 healthy controls were included, their meta-analysis showed that there were some important group differences in the changes from Day 1 to Day 2. Despite similar reduction in workload as controls on Day 2, the patients displayed a larger decline in oxygen consumption the second day compared with their measures on Day 1. They also found that the patients had a clearly reduced workload at the point when hyperventilation starts compared with controls. This suggests an earlier onset of anaerobic metabolism among the patients on Day 2, which is consistent with the finding of reduced workload for the onset of blood lactate on Day 2 (Lien et al., 2019). These observations can hardly be used for diagnostic purposes but suggest that the first day of exercise induces an altered metabolism in patients the following days. To what extent the altered responses are generalized or restricted to the exercising muscles, is unknown. However, these observations suggest that the CPET exercise model may be valuable in further investigations of metabolism in ME/CFS.

Muscular metabolism during exercise depends heavily on cardiovascular responses. Nelson and co-workers made a review and meta-analysis of heart rate parameters in relation to exercise and other triggers (Nelson et al., 2019). They found that patients with ME/CFS had deviations in numerous heart rate parameters, suggesting reduced vagal and increased sympathetic modulation of heart rate. A recent study of heart rate parameters measured before, during and after exercise indicate diminished cardiac parasympathetic and sympathetic activation during supine rest and exercise in patients with ME/CFS (Van Oosterwijck et al., 2021). Furthermore, they observed a reduced parasympathetic reactivation after exercise among patients compared with controls. In blood samples obtained before, during and after exercise, Strahler and coworkers found decreased level of adrenaline during exercise in patients (Strahler et al., 2013). In samples taken before or 30 min after exercise, the levels were not different from healthy controls.

Taken together, the studies of repeated CPETs indicate that the muscular metabolism and autonomic response are altered in patients with ME/CFS. Furthermore, exercise appears to induce a prolonged decrease in muscular force or maximal workload. These responses may correspond to patients’ experiences of increased exertion.

## Discussion

It is widely accepted that PEM is a hallmark of ME/CFS and that a broad range of events or activities can trigger PEM. The patients describe PEM with a wide variety of words and report that it lasts long, even up to months. This variation leads to challenges when comparing and synthesizing patients’ experiences and expressions of PEM across patients and studies. As pointed out by Jason and co-workers slight differences in wording may have an impact on the results and conclusions (Jason et al., 2015b). Furthermore, differentiating between tiredness, fatigue and exhaustion may be of importance. This is of relevance for the qualitative as well as the questionnaire-based studies. Furthermore, the studies rarely define what they mean by PEM. It seems that the respondents are expected to have *a priori* understanding of the phenomenon. One way to go might be to distinguish more clearly between triggers that patients have in terms of load from their daily activities and extraordinary exertions such as intense bouts of exercise. Another approach might be to emphasize and discriminate between the two domains of PEM (generalized and muscular fatigue) or the three levels used by patients (tiredness, fatigue, and exhaustion) in future studies.

So far, research has not led to identification of underlying mechanisms of PEM. Interestingly, PEM is not unique and specific for ME/CFS but is quite frequent also in other chronic diseases (Nacul et al., 2021). Studies show relatively high prevalence of PEM-like symptoms in for instance persons with multiple sclerosis or long COVID (Morris and Maes, 2013; Learmonth et al., 2014; Hodges et al., 2018; Jason and Dorri, 2022). The descriptors used by other patient groups than ME/CFS are also similar, but the concept of PEM is not coined to the disease and not part of the diagnostic criteria for the other diseases. The rapidly emerging knowledge about individuals with long COVID symptoms may be of particular interest. These patients report similar perceptions of fatigue as patients with ME/CFS as well as some of the same indications of altered exercise responses (Wong and Weitzer, 2021; Joseph et al., 2023). It has been argued that the altered exercise responses may be caused by deconditioning (Rinaldo et al., 2021) and a recent brief report indicates that the altered responses may be normalized within 2 years (Rinaldo et al., 2023). Hence, also for individuals with long COVID symptoms, it is still uncertain whether the reported deviations in exercise responses is connected to the clinical condition and the symptoms, or if they can be simply explained by deconditioning (Durstenfeld et al., 2022).

It is also intriguing that symptoms very similar to PEM are typical for overtraining syndrome of athletes (Meeusen et al., 2013). Similarly, to patients with ME/CFS, they report elevated perceived exertion and fatigue during exercise. However, the magnitude of the responses is somewhat lower, and PEM attenuates faster. The causes of these differences are unknown, but a possible explanation may be related to the degree of severity of fatigue or disease. Overtrained athletes also show attenuated responses in adrenaline and noradrenaline and thus indicate altered neuroendocrinological responses (Armstrong et al., 2021). The similarities across various groups and conditions may be useful in further examination and research of underlying nature of PEM as distinct from everyday responses to activities.

The finding that PEM may be composed of two different constructs, generalized fatigue and muscle fatigue (McManimen et al., 2019), could be an important clue to enhance our understanding of PEM. An obvious hypothesis is that the self-reported muscle fatigue of PEM is closely related to a decrease in the muscular strength, which might be measured as maximal force. It is well known that the ability to generate force declines during an exercise session (Vøllestad, 1997). This response may be due to changes in the muscles capacity to generate force (muscular mechanisms), or it may be due to a decreased ability to drive the muscle to its full capacity (motor control mechanisms). It seems important that these different responses are studied also in ME/CFS following exercise, to help in understanding the mechanisms involved in at least the muscular component of PEM. It would also be of interest to compare the responses of not only ME/CFS and healthy controls but include other chronic diseases and overtraining syndrome of athletes. Furthermore, to our knowledge, no one has examined if for instance the presence or level of the two PEM constructs (muscle-specific fatigue and generalized fatigue) are closely linked. Or could it be that the construct of muscle-specific fatigue is particularly triggered by physical exertion, with less impact on generalized fatigue?

There are two striking post-exercise differences between patients with ME/CFS and healthy controls: The patients have a higher prevalence of symptoms and higher intensity of symptoms in the days following exercise. It is interesting, and somewhat surprising, that we have limited data on biological factors and processes in the restitution phase. The repeated CPETs performed on successive days show some deviations (e.g., in workload and lactate accumulation) in the exercise responses on Day 2 (Lien et al., 2019; Hodges et al., 2020). Thus, there is a need to conduct more comprehensive studies to examine for instance post-exercise changes in autonomic responses and in the ability of muscles to generate force. Apart from the studies employing repeated CPETs, the exercise models have predominantly focused on self-reported symptoms in the post-exercise period. Based on the patients’ descriptions of days and weeks to recover from exercise, research of physiological responses should probably be extended to capture the restitution phase. The focus could then shift from what triggers PEM to why does PEM triggered by exercise show a slower restitution in ME/CFS patients than in healthy controls.

As shown above the symptoms described by patients with ME/CFS may take different forms and be expressed in different ways. From the words used by the patients, it seems that the word “tired” or “tiredness” could be used to describe a normal response to an exertion, experienced by healthy persons. In a situation when ME/CFS has been established, “fatigue” seems to be a common term to denote the every-day experience without prior triggers (Olson et al., 2015). When these persons exercise, they experience a combination of fatigue and tiredness, while PEM is the prolonged exacerbation seen in the post-exercise period. Such distinctions between the terms for fatigue, may create a better framework for research and clinical communication.

The forms and descriptions of triggers of PEM vary as well. Since patients with ME/CFS typically reduce their physical activity level, their reference frame will often be daily life and triggers include for instance household chores and social activities (Stussman et al., 2020). Yet, the research searching for biological causes of PEM, uses intense exercise such as CPET as triggers. This may restrict our knowledge generation to the responses of extraordinary exertions, with uncertain relevance for understanding PEM induced by daily activities. It is also important to emphasize that studies including exercise models, and in particular CPET, require eligible participants that are able to complete the expected work. Hence, these models will probably include a selected group of patients, either with a lighter disease burden or patients in a recovery phase. Furthermore, the kind of symptoms studied in the post exertional period to assess PEM, are rather narrow compared with the wide spectrum expressed by the patients in interviews. We believe these aspects and possible limitations of experimental exercise tests need attention in future research.

In summary, there are some indications that exercise induces symptoms and metabolic or neuroendocrine responses in patients with ME/CFS that differ from what is seen in healthy individuals. However, similar deviations are also reported for other patient groups and overtrained athletes. The relevance of these deviations for case definition and diagnostic purposes thus needs further investigation. To enhance our understanding of underlying mechanisms of PEM or ME/CFS as a disease, we believe it is particularly important to look more closely into the processes occurring in the restitution period following exercise tests, as PEM reaches the peak in this phase.

## Data Availability

The original contributions presented in the study are included in the article/Supplementary Material, further inquiries can be directed to the corresponding author.

## References

[B1] Adachi H. (2017). Cardiopulmonary exercise test. Int. Heart J. 58 (5), 654–665. 10.1536/ihj.17-264 28966333

[B2] Armstrong L. E. Bergeron M. F. Lee E. C. Mershon J. E. Armstrong E. M. (2021). Overtraining syndrome as a complex systems phenomenon. Front. Netw. Physiol. 1, 794392. 10.3389/fnetp.2021.794392 36925581 PMC10013019

[B3] Åstrand P. O. Rodahl K. Dahl H. A. Strømme S. B. (2003). Textbook of work Physiology. Physiological bases of exercise. 4th ed. Champaign, Ill: Human Kinetics.

[B4] Baraniuk J. N. Adewuyi O. Merck S. J. Ali M. Ravindran M. K. Timbol C. R. (2013). A Chronic Fatigue Syndrome (CFS) severity score based on case designation criteria. Am. J. Transl. Res. 5 (1), 53–68. 23390566 PMC3560481

[B5] Barhorst E. E. Andrae W. E. Rayne T. J. Falvo M. J. Cook D. B. Lindheimer J. B. (2020). Elevated perceived exertion in people with myalgic encephalomyelitis/chronic fatigue syndrome and fibromyalgia: a meta-analysis. Med. Sci. Sports Exerc 52 (12), 2615–2627. 10.1249/mss.0000000000002421 32555018 PMC10200687

[B6] Barhorst E. E. Boruch A. E. Cook D. B. Lindheimer J. B. (2022). Pain-related post-exertional malaise in myalgic encephalomyelitis/chronic fatigue syndrome (ME/CFS) and fibromyalgia: a systematic review and three-level meta-analysis. Pain Med. 23 (6), 1144–1157. 10.1093/pm/pnab308 34668532

[B7] Brown A. A. Jason L. A. (2014). Validating a measure of myalgic encephalomyelitis/chronic fatigue syndrome symptomatology. Fatigue Biomed. Health & Behav. 2 (3), 132–152. 10.1080/21641846.2014.928014 PMC4871625 27213118

[B8] Carruthers B. M. van de Sande M. I. De Meirleir K. L. Klimas N. G. Broderick G. Mitchell T. (2011). Myalgic encephalomyelitis: international consensus criteria. J. Intern Med. 270 (4), 327–338. 10.1111/j.1365-2796.2011.02428.x 21777306 PMC3427890

[B9] Chu L. Valencia I. J. Garvert D. W. Montoya J. G. (2018). Deconstructing post-exertional malaise in myalgic encephalomyelitis/chronic fatigue syndrome: a patient-centered, cross-sectional survey. Plos One 13 (6), e0197811. 10.1371/journal.pone.0197811 29856774 PMC5983853

[B10] Cook D. B. VanRiper S. Dougherty R. J. Lindheimer J. B. Falvo M. J. Chen Y. (2022). Cardiopulmonary, metabolic, and perceptual responses during exercise in myalgic encephalomyelitis/chronic fatigue syndrome (ME/CFS): a multi-site clinical assessment of ME/CFS (mcam) sub-study. PLoS One 17 (3), e0265315. 10.1371/journal.pone.0265315 35290404 PMC8923458

[B11] Cotler J. Holtzman C. Dudun C. Jason L. A. (2018). A brief questionnaire to assess post-exertional malaise. Diagn. (Basel) 8 (3), 66. 10.3390/diagnostics8030066 PMC6165517 30208578

[B12] Davenport T. E. Chu L. Stevens S. R. Stevens J. Snell C. R. Van Ness J. M. (2023b). Two symptoms can accurately identify post-exertional malaise in myalgic encephalomyelitis/chronic fatigue syndrome. Work 74 (4), 1199–1213. 10.3233/wor-220554 36938769

[B13] Davenport T. E. Stevens S. R. Stevens J. Snell C. R. Van Ness J. M. (2023a). Development and measurement properties of the PEM/PESE activity questionnaire (PAQ). Work 74 (4), 1187–1197. 10.3233/WOR-220553 36938768

[B14] Durstenfeld M. S. Sun K. Tahir P. Peluso M. J. Deeks S. G. Aras M. A. (2022). Use of cardiopulmonary exercise testing to evaluate long COVID-19 symptoms in adults: a systematic review and meta-analysis. JAMA Netw. Open 5 (10), e2236057. 10.1001/jamanetworkopen.2022.36057 36223120 PMC9557896

[B15] Fukuda K. Straus S. E. Hickie I. Sharpe M. C. Dobbins J. G. Komaroff A. (1994). The chronic fatigue syndrome: a comprehensive approach to its definition and study. International Chronic Fatigue Syndrome Study Group. Ann. Intern Med. 121 (12), 953–959. 10.7326/0003-4819-121-12-199412150-00009 7978722

[B16] Hartle M. Bateman L. Vernon S. D. (2021). Dissecting the nature of post-exertional malaise. Fatigue (Abingdon, Eng. 9 (1), 33–44. 10.1080/21641846.2021.1905415

[B17] Hodges L. Nielsen T. Cochrane D. Baken D. (2020). The physiological time line of post-exertional malaise in Myalgic Encephalomyelitis/Chronic Fatigue Syndrome (ME/CFS). Transl. Sports Med. 3 (3), 243–249. 10.1002/tsm2.133

[B18] Hodges L. D. Nielsen T. Baken D. (2018). Physiological measures in participants with chronic fatigue syndrome, multiple sclerosis and healthy controls following repeated exercise: a pilot study. Clin. Physiol. Funct. Imaging 38 (4), 639–644. 10.1111/cpf.12460 28782878

[B19] Holtzman C. S. Bhatia S. Cotler J. Jason L. A. (2019). Assessment of post-exertional malaise (PEM) in patients with myalgic encephalomyelitis (ME) and chronic fatigue syndrome (CFS): a patient-driven survey. Diagn. (Basel) 9 (1), 26. 10.3390/diagnostics9010026 PMC6468435 30832336

[B20] Jason L. A. Dorri J. A. (2022). ME/CFS and post-exertional malaise among patients with long COVID. Neurol. Int. 15 (1), 1–11. 10.3390/neurolint15010001 36648965 PMC9844405

[B21] Jason L. A. Evans M. So S. Scott J. Brown A. (2015b). Problems in defining post-exertional malaise. J. Prev. Interv. Community 43 (1), 20–31. 10.1080/10852352.2014.973239 25584525 PMC4295644

[B22] Jason L. A. McManimen S. L. Sunnquist M. Holtzman C. S. (2018). Patient perceptions of post exertional malaise. Fatigue Biomed. Health & Behav. 6 (2), 92–105. 10.1080/21641846.2018.1453265

[B23] Jason L. A. Sunnquist M. (2018). The development of the DePaul symptom questionnaire: original, expanded, brief, and pediatric versions. Front. Pediatr. 6, 330. 10.3389/fped.2018.00330 30460215 PMC6232226

[B24] Jason L. A. Sunnquist M. Brown A. Furst J. Cid M. Farietta J. (2015a). Factor analysis of the DePaul symptom questionnaire: identifying core domains. J. Neurol. Neurobiol. 1 (4). 10.16966/2379-7150.114 PMC4830389 27088131

[B25] Joseph P. Singh I. Oliveira R. Capone C. A. Mullen M. P. Cook D. B. (2023). Exercise pathophysiology in myalgic encephalomyelitis/chronic fatigue syndrome and postacute sequelae of SARS-CoV-2: more in common than not? Chest 164 (3), 717–726. 10.1016/j.chest.2023.03.049 37054777 PMC10088277

[B26] Krabbe S. H. Mengshoel A. M. Schroder Bjorbaekmo W. Sveen U. Groven K. S. (2022). Bodies in lockdown: young women's narratives of falling severely ill with ME/CFS during childhood and adolescence. Health Care Women Int. 2022, 1–23. 10.1080/07399332.2022.2043862 35404768

[B27] Learmonth Y. C. Paul L. McFadyen A. K. Marshall-McKenna R. Mattison P. Miller L. (2014). Short-term effect of aerobic exercise on symptoms in multiple sclerosis and chronic fatigue syndrome: a pilot study. Int. J. MS Care 16 (2), 76–82. 10.7224/1537-2073.2013-005 25061431 PMC4106394

[B28] Lien K. Johansen B. Veierød M. B. Haslestad A. S. Bøhn S. K. Melsom M. N. (2019). Abnormal blood lactate accumulation during repeated exercise testing in myalgic encephalomyelitis/chronic fatigue syndrome. Physiol. Rep. 7 (11), e14138. 10.14814/phy2.14138 31161646 PMC6546966

[B29] Lim E. J. Kang E. B. Jang E. S. Son C. G. (2020). The prospects of the two-day cardiopulmonary exercise test (CPET) in ME/CFS patients: a meta-analysis. J. Clin. Med. 9 (12), 4040. 10.3390/jcm9124040 33327624 PMC7765094

[B30] Locher L. IOM (2015). Beyond myalgic encephalomyelitis/chronic fatigue syndrome: redefining an illness. Washington, District of Columbia: The National Academies Press. 25695122

[B31] Loy B. D. O'Connor P. J. Dishman R. K. (2016). Effect of acute exercise on fatigue in people with ME/CFS/SEID: a meta-analysis. Med. Sci. Sports Exerc 48 (10), 2003–2012. 10.1249/MSS.0000000000000990 27187093 PMC5026555

[B32] May M. Milrad S. F. Perdomo D. M. Czaja S. J. Fletcher M. A. Jutagir D. R. (2020). Post-exertional malaise is associated with greater symptom burden and psychological distress in patients diagnosed with Chronic Fatigue Syndrome. J. Psychosom. Res. 129, 109893. 10.1016/j.jpsychores.2019.109893 31884303 PMC7007968

[B33] McManimen S. L. Sunnquist M. L. Jason L. A. (2019). Deconstructing post-exertional malaise: an exploratory factor analysis. J. Health Psychol. 24 (2), 188–198. 10.1177/1359105316664139 27557649 PMC5325824

[B34] Meeusen R. Duclos M. Foster C. Fry A. Gleeson M. Nieman D. (2013). Prevention, diagnosis, and treatment of the overtraining syndrome: joint consensus statement of the European College of Sport Science and the American College of Sports Medicine. Med. Sci. Sports Exerc 45 (1), 186–205. 10.1249/MSS.0b013e318279a10a 23247672

[B35] Moore G. E. Keller B. A. Stevens J. Mao X. Stevens S. R. Chia J. K. (2023). Recovery from exercise in persons with myalgic encephalomyelitis/chronic fatigue syndrome (ME/CFS). Med. Kaunas. 59 (3), 571. 10.3390/medicina59030571 PMC10059925 36984572

[B36] Morris G. Maes M. (2013). Myalgic encephalomyelitis/chronic fatigue syndrome and encephalomyelitis disseminata/multiple sclerosis show remarkable levels of similarity in phenomenology and neuroimmune characteristics. BMC Med. 11, 205. 10.1186/1741-7015-11-205 24229326 PMC3847236

[B37] Nacul L. Authier F. J. Scheibenbogen C. Lorusso L. Helland I. B. Martin J. A. (2021). European network on myalgic encephalomyelitis/chronic fatigue syndrome (EUROMENE): expert consensus on the diagnosis, service provision, and care of people with ME/CFS in europe. Med. Kaunas. 57 (5), 510. 10.3390/medicina57050510 PMC8161074 34069603

[B38] Nelson M. J. Bahl J. S. Buckley J. D. Thomson R. L. Davison K. (2019). Evidence of altered cardiac autonomic regulation in myalgic encephalomyelitis/chronic fatigue syndrome: a systematic review and meta-analysis. Med. Baltim. 98 (43), e17600. 10.1097/md.0000000000017600 PMC6824690 31651868

[B39] NICE (2021). Myalgic encephalomyelitis (or encephalopathy)/chronic fatigue syndrome: diagnosis and management. NICE guideline [NG206]. Available at: www.https://www.nice.org.uk/guidance/ng206/resources (Accessed October 29, 2021) 35438859

[B40] Olson K. Zimka O. Stein E. (2015). The nature of fatigue in chronic fatigue syndrome. Qual. Health Res. 25 (10), 1410–1422. 10.1177/1049732315573954 25721719

[B41] Oosterwijck J. V. Marusic U. De Wandele I. Paul L. Meeus M. Moorkens G. (2017). The role of autonomic function in exercise-induced endogenous analgesia: a case-control study in myalgic encephalomyelitis/chronic fatigue syndrome and healthy people. Pain Physician 20 (3), E389–E399. 28339438

[B42] Parslow R. M. Anderson N. Byrne D. Shaw A. Haywood K. L. Crawley E. (2018). Adolescent's descriptions of fatigue, fluctuation and payback in chronic fatigue syndrome/myalgic encephalopathy (CFS/ME): interviews with adolescents and parents. BMJ Paediatr. Open 2 (1), e000281. 10.1136/bmjpo-2018-000281 PMC6307594 30613800

[B43] Rinaldo R. F. Mondoni M. Baccelli A. Marchetti F. Re B. Degrassi M. (2023). SARS-CoV-2 infection sequelae on exercise response: persistent or reversible? A 2-year perspective. ERJ Open Res. 9 (4), 00234-2023. 10.1183/23120541.00234-2023 37389900 PMC10291727

[B44] Rinaldo R. F. Mondoni M. Parazzini E. M. Pitari F. Brambilla E. Luraschi S. (2021). Deconditioning as main mechanism of impaired exercise response in COVID-19 survivors. Eur. Respir. J. 58 (2), 2100870. 10.1183/13993003.00870-2021 33926969 PMC8082950

[B45] Saltin B. Blomqvist G. Mitchell J. H. Johnson R. L. Jr. Wildenthal K. Chapman C. B. (1968). A longitudinal study of adaptive changes in oxygen transport and body composition. Circulation 38 (5), Vii1–78. 10.1161/01.cir.38.5s7.vii-1 5696236

[B46] Sandler C. X. Lloyd A. R. Barry B. K. (2016). Fatigue exacerbation by interval or continuous exercise in chronic fatigue syndrome. Med. Sci. Sports Exerc 48 (10), 1875–1885. 10.1249/mss.0000000000000983 27183124

[B47] Sharpe M. C. Archard L. C. Banatvala J. E. Borysiewicz L. K. Clare A. W. David A. (1991). A report--chronic fatigue syndrome: guidelines for research. J. R. Soc. Med. 84 (2), 118–121. 10.1177/014107689108400224 1999813 PMC1293107

[B48] Strahler J. Fischer S. Nater U. M. Ehlert U. Gaab J. (2013). Norepinephrine and epinephrine responses to physiological and pharmacological stimulation in chronic fatigue syndrome. Biol. Psychol. 94 (1), 160–166. 10.1016/j.biopsycho.2013.06.002 23770415

[B49] Strand E. B. Mengshoel A. M. Sandvik L. Helland I. B. Abraham S. Nes L. S. (2019). Pain is associated with reduced quality of life and functional status in patients with Myalgic Encephalomyelitis/Chronic Fatigue Syndrome. Scand. J. Pain 19 (1), 61–72. 10.1515/sjpain-2018-0095 30325738

[B50] Strassheim V. Newton J. L. Collins T. (2021). Experiences of living with severe chronic fatigue syndrome/myalgic encephalomyelitis. Healthc. (Basel). 9 (2), 168. 10.3390/healthcare9020168 PMC7914910 33562474

[B51] Stussman B. Williams A. Snow J. Gavin A. Scott R. Nath A. (2020). Characterization of post-exertional malaise in patients with myalgic encephalomyelitis/chronic fatigue syndrome. Front. Neurol. 11, 1025. 10.3389/fneur.2020.01025 33071931 PMC7530890

[B52] Van Oosterwijck J. Marusic U. De Wandele I. Meeus M. Paul L. Lambrecht L. (2021). Reduced parasympathetic reactivation during recovery from exercise in myalgic encephalomyelitis/chronic fatigue syndrome. J. Clin. Med. 10 (19), 4527. 10.3390/jcm10194527 34640544 PMC8509376

[B53] Vøllestad N. K. (1997). Measurement of human muscle fatigue. J. Neurosci. Methods 74 (2), 219–227. 10.1016/s0165-0270(97)02251-6 9219890

[B54] Wong T. L. Weitzer D. J. (2021). Long COVID and myalgic encephalomyelitis/chronic fatigue syndrome (ME/CFS)-A systemic review and comparison of clinical presentation and symptomatology. Med. Kaunas. 57 (5), 418. 10.3390/medicina57050418 PMC8145228 33925784

